# Population preference values for treatment outcomes in chronic lymphocytic leukaemia: a cross-sectional utility study

**DOI:** 10.1186/1477-7525-8-50

**Published:** 2010-05-18

**Authors:** Kathleen M Beusterien, John Davies, Michael Leach, David Meiklejohn, Jessica L Grinspan, Alison O'Toole, Steve Bramham-Jones

**Affiliations:** 1 Oxford Outcomes, Bethesda, MD, USA; 2 Western General Hospital, Edinburgh, UK; 3 Leukaemia Research Lab, Glasgow, UK; 4 NHS Tayside, Dundee, UK; 5 Napp Pharmaceuticals Limited, Cambridge, UK

## Abstract

**Background:**

Given that treatments for chronic lymphocytic leukaemia (CLL) are palliative rather than curative, evaluating the patient-perceived impacts of therapy is critical. To date, no utility (preference) studies from the general public or patient perspective have been conducted in CLL. The objective of this study was to measure preferences for health states associated with CLL treatment.

**Methods:**

This was a cross-sectional study of 89 members of the general population in the UK (England and Scotland). Using standard gamble, each participant valued four health states describing response status, six describing treatment-related toxicities based on Common Toxicity Criteria, and two describing line of treatment. The health states incorporated standardized descriptions of treatment response (symptoms have "improved," "stabilized," or "gotten worse"), swollen glands, impact on daily activities, fatigue, appetite, and night sweats. Utility estimates ranged from 0.0, reflecting dead, to 1.0, reflecting full health.

**Results:**

Complete response (CR) was the most preferred health state (mean utility, 0.91), followed by partial response (PR), 0.84; no change (NC), 0.78; and progressive disease (PD), 0.68. Among the toxicity states, grade I/II nausea and nausea/vomiting had the smallest utility decrements (both were -0.05), and grade III/IV pneumonia had the greatest decrement (-0.20). The utility decrements obtained for toxicity states can be subtracted from utilities for CR, PR, NC, and PD, as appropriate. The utilities for second- and third-line treatments, which are attempted when symptoms worsen, were 0.71 and 0.65, respectively. No significant differences in utilities were observed by age, sex, or knowledge/experience with leukaemia.

**Conclusions:**

This study reports UK population utilities for a universal set of CLL health states that incorporate intended treatment response and unintended toxicities. These utilities can be applied in future cost-effectiveness analyses of CLL treatment.

## Background

Chronic lymphocytic leukaemia (CLL) is a progressive form of leukaemia characterised by an accumulation of abnormal lymphocytes that have lost the ability to undergo apoptosis (programmed cell death). These lymphocytes accumulate in the lymph nodes, liver, spleen, blood, and bone marrow and compromise the activity of cell mediated and humoral immunity with loss of immune memory. Consequently, patients with CLL typically experience recurrent infections, some of which can be serious [1]. CLL predominantly is a disease among older individuals, with a median age at diagnosis of 72 years [2]. Patient age, however, does not appear to influence the presence of symptoms, with no difference in clinical findings found between younger (<55) and older patient populations [1]. CLL is approximately twice as common in men as in women, and it is the most common form of leukaemia in Western nations, with an annual incidence of 3 to 3.5 cases per 100,000 [2].

With the exception of blood and marrow transplantation, CLL is an inherently incurable condition and treatments are therefore focused on controlling symptoms and optimising health-related quality of life [3]. Thus, quantifying the patient-perceived impact of therapy is a critical outcome in CLL research. Health status utility assessments enable quantification of preferences for selected health outcomes and, consequently, estimation of quality-adjusted life years (QALYs). Health technology assessment (HTA) agencies established by regulatory authorities in various countries such as the United Kingdom, Australia, and Canada emphasize the importance of QALYs in product evaluation. For example, a briefing paper disseminated by the National Institute for Health and Clinical Excellence (NICE) cites that health state utility values are a key parameter in cost-effectiveness models and have been found to have a major impact on the results of many appraisals [4]. As the Scottish Medicines Consortium (SMC) states, they have a preference for cost-utility analyses using QALYs as the primary outcome measure in order to make clear comparisons of the value of new medicines [5]. QALYs are particularly useful for conditions for which treatment is not curative but palliative, such as cancer. For example, the incremental cost per QALY gained was a key endpoint in recent cost-effectiveness evaluations in CLL and chronic myeloid leukaemia (CML) [6-8].

To date, no utility studies from the patient or general population perspective have been conducted in CLL [9,10]. While one clinician group, the Wessex Development and Evaluation Committee (1995), attempted to estimate utility weights for select CLL health states by gauging where patients might be on the Index of Health-related Quality of Life (IHQOL) measure, researchers have since recommended caution in using utilities based on such simplistic methodology [11,12]. Thus, there is an unmet need for a *de novo *utility study in CLL.

HTA agencies generally prefer that generic measures such as the EQ-5D are used to estimate utilities incorporated into cost-effectiveness evaluations [4]. However, generic measures, as opposed to disease-specific utility measures, may not adequately capture key psychological impacts associated with CLL treatment, for example, the impact of knowing that one is responding to treatment. This knowledge, and the resulting hopefulness that may result, can have substantial impact on a patient's outlook and psychological health. For example, in a qualitative study of patients with chronic leukaemia that investigated how these patients conceptualise quality of life, hope was identified as a key theme associated with coping [13].

Little work in the area of preference-based utilities has been conducted that captures both the intended clinical response and unintended toxicities associated with treatment. Measurement of preferences for health states characterizing cancer-specific states associated with treatment can be particularly valuable in order to help clinicians and decision-makers better understand the balance between the physiological and psychological benefits of treatment versus the negative impact of these treatments on daily life [14]. The purpose of the current study was to use a vignette-based utility approach to measure preferences for standardized health states that include clinical response and toxicities observed during treatment of CLL.

## Methods

A cross-sectional study was performed to elicit utilities for CLL health states among, as suggested by the UK National Institute for Health and Clinical Excellence, members of the general public [4]. Four trained interviewers used the standard gamble technique, the only utility technique consistent with the axioms of utility theory, which involves making decisions under conditions of uncertainty [15]. Respondents imagine that they are in a selected health state. They can remain in that state, or take a gamble that involves a chance (*p*) of achieving full health with a corresponding chance (1-*p*) of being dead. The *p *probabilities are varied using a ping-pong approach until the respondent is indifferent between the two options. The interviewer used a chance board with a probability wheel using 2-color pie charts to illustrate the different probabilities.

Study participants were recruited from the general population in the UK, including both England and Scotland, in March 2009. Each interviewer recruited 15-25 laypeople in the U.K. (England and Scotland) through word-of-mouth as well as through a panel of laypeople who had previously volunteered to be participants for research studies. Eligible participants were residents of England or Scotland, aged 18 or over, and capable of giving informed consent. The interviews were conducted in-person, and the participants completed a demographic questionnaire and, prior to beginning the standard gamble exercise, they were asked to order the health states from most preferable to least preferable. All participants provided informed consent and received compensation of 25GBP for their time. This study was approved by the Independent Investigational Review Board (Plantation, FL) and complied with the tenets of the Declaration of Helsinki.

### Health State Development

The health states were designed to describe the functional and patient-centred impacts of CLL and it treatment, rather than clinical descriptions of the disease, in line with published guidelines for health state development [15]. Development was an iterative process that involved incorporating input from the literature, patient web-based discussion forums, physicians with expertise in CLL, and patients with CLL.

The following domains were described, enabling balanced descriptions across the health states: cancer description, "cancer of the blood"; treatment response category; swollen glands in neck, armpits, or groin; limitations in performing daily activities; level of fatigue; appetite; and trouble sleeping because of night sweats. These domains are those that previous researchers have identified as important in CLL. In a review of the burden and outcomes associated with leukaemia, for example, Redaelli *et al *[16] reported that physical functioning, role-functioning, and fatigue/energy levels were among the most important domains related to CLL. With regard to symptoms, Kermani *et al *[17] found that, in CLL patients, fatigue was the most common (present in 54% of patients), followed by dyspnoea (32%), abdominal pain (29%), and weight loss (22%). In addition, lymphadenopathy (enlarged lymph nodes in the groin, spleen, or neck) was observed in 89% of patients. Other researchers [18,19] also have reported that, compared to population norms, CLL patients have more complaints about fatigue, appetite loss, and sleep disturbances. Based on patient posts on the web-based CLL forums, frequent and bothersome impacts of CLL were related to fatigue/energy levels, ability to perform daily activities, appetite disturbances, sleep disturbances, and noticeably enlarged lymph nodes.

In all, four CLL treatment-related response states, six toxicity-related health states, and two health states reflecting the impact of undergoing a second or third course of treatment were developed (health states located in Additional File 1). The domains describing clinical response status were based on the National Cancer Institute Working Group (NCIWG)'s treatment response definitions for CLL [20]. The *complete response *state was based on the complete absence of symptoms; *partial response *represented a ≥ 50% reduction in symptoms; *no change *meant that the disease was stable (i.e., symptoms not worsening or improving); and *progressive disease *indicated that symptoms were worsening.

With respect to the toxicity states, these were identified based on common toxicities experienced with bendamustine and chlorambucil, and draft descriptions were developed using the Cancer Therapy Evaluation program's Common Terminology Criteria version 3.0 [21]. Because it would be too cumbersome for respondents to value all possible combinations of clinical response status with the various possible toxicities, the toxicity health states described each toxicity in association with the base health state of *no change *(NC) health state. NC was selected to pair with the toxicities as opposed to PR given that this is a more conservative approach; if the toxicities were coupled with PR, for example, it is possible that respondents would not rate them as poorly as they would if they were coupled with NC because they may be more accepting of an aggressive therapy if they know that the treatment is working.

The draft health states were refined after iterative review by four independent clinicians. Draft health states were tested in five Scottish CLL patients recruited through the CLL Support Association, a web-based support group based in the UK, and final revisions were made based on their feedback. The states were developed to be easily comprehensible from the general public's perspective and gender-neutral. During the interview, the health states were labelled using symbols, as opposed to numerical identifiers, to avoid imposing any hierarchical order among them.

### Statistical Analysis

This study was designed to collect data from 93 people; this was not determined or based on a formal power analysis because there was no specific hypothesis to test. Demographic data were summarised by means and standard deviations for continuous variables, and proportions for categorical variables. Means were calculated for the rank orderings of the health states. For each health state, the respective standard gamble utility equalled the probability *p *of full health at the point where the respondent was indifferent between staying in the health state and taking the gamble. Utility scores range from 0.0, reflecting being dead, to 1.0, reflecting full health. A decision rule was implemented for eliminating illogical responses. Specifically, participants who had at least three illogical responses (e.g., valuing *no change *plus a toxicity as higher than *no change*) were eliminated from all analyses. Utilities were summarized using means, standard deviations, medians, 95% confidence intervals (95% CI), and standard errors (SE). Utility decrements for toxicities were generated by subtracting the utility for the base case (*no change*) from the utility of the toxicity state (each of which was described in association with *no change*). Although the study was not powered to test for differences in utilities between subgroups, exploratory analyses were made comparing utilities by region (England vs. Scotland), age group, sex, and whether or not the respondents were knowledgeable about leukaemia using the Student's t-test. All statistical analyses were conducted using SAS v9.0.

## Results

In total, 93 respondents were recruited from the UK, including 62 from England and 31 from Scotland. Of these, four respondents (4.5%) provided at least three illogical responses (utility weights were higher for a less favourable health state versus a more favourable health state), and they were excluded from the analysis. Thus, 89 participants, 59 from England and 30 from Scotland, were included in the final analysis. In comparison to the demographic distributions of the target adult populations in Scotland and in England & Wales based on the 2001 UK census [22], fewer individuals in the study sample were from ages 18-24 (8% vs. 14%) and more had qualifications or achieved greater than Level 1 education (80% vs. 71%). Mean respondent age was 47 ± 16 years, and 44% were male. The respondents represented five cities in Scotland and nine cities in England.

As expected, *full health *and *complete response *had the first and second highest mean rankings, respectively, among the health states, followed by *partial response *and no change, respectively. The grade 1/2 toxicity health states were ranked higher than the grade 3/4 toxicity health states, and *no change plus grade 3/4 pneumonia *was ranked the worst.

Table 1 reports the standard gamble utility values for the CLL health states for the total sample. As expected, among the clinical response states, *complete response *was most preferred (utility = 0.91), followed by *partial response *(utility = 0.84), followed by *no change *(utility = 0.78) and *progressive disease *(utility = 0.68), respectively. The number of treatment attempts required in CLL (first, second, or third-line therapy) was associated with different utility weights. Specifically, the utility for *second-line therapy *(0.71) was lower than that found for *no change *(0. 78), and the utility for *third-line treatment *was lower than that of progressive disease (0.68).

**Table 1:** Mean utility values for UK general public (N = 89).

Health State	Mean ± SD	95% CI (lower, upper)	Toxicity disutility
Complete Response	0.91 ± 0.11	0.88, 0.93	NA

Partial Response	0.84 ± 0.14	0.81, 0.87	NA

No Change	0.78 ± 0.14	0.75, 0.82	NA

NC + 1-2 Nausea	0.73 ± 0.17	0.69, 0.76	-0.05 (0.02)

NC + 1-2 Nausea/Vomiting	0.73 ± 0.16	0.69, 0.76	-0.05 (0.02)

Second-line Treatment	0.71 ± 0.17	0.68, 0.75	NA

NC + 1-2 Diarrhea	0.70 ± 0.19	0.66, 0.74	-0.08 (0.02)

NC + 3-4 Anemia	0.69 ± 0.18	0.65, 0.72	-0.09 (0.02)

Progressive Disease	0.68 ± 0.20	0.64, 0.72	NA

NC + 3-4 Pyrexia	0.67 ± 0.17	0.63, 0.70	-0.11 (0.02)

Third-line Treatment	0.65 ± 0.22	0.60, 0.69	NA

NC + 3-4 Pneumonia	0.58 ± 0.19	0.54, 0.62	-0.20 (0.02)

The health states that comprised *no change *plus toxicities were less preferred than the *no change *base state, and the *no change *plus grade 3/4 (more severe) toxicity health states had lower utilities than the *no change *plus grade 1/2 (less severe) toxicity health states. Of all of the health states, *no change plus grade 3/4 pneumonia *was the least preferred (utility = 0.58). Table 1 reports the respective utility decrements associated with each toxicity state. These can be added to those values for the *complete response, partial response*, *progressive disease*, and *no change*, as applicable.

A comparison of utility data between England and Scotland showed that utility weights reported by Scottish respondents were higher than those reported by English participants for most of the health states (Table 2). Nevertheless, the relative differences between health states were generally similar between the regions, and thus the decrements associated with the various toxicity states were comparable between the English and Scottish samples. No significant differences in utilities were observed between older (≥ 60 years) versus younger patients, between males versus females, or between higher versus lower educational levels achieved (Levels 0-2 vs. Levels 3-5); however, the study was not powered to detect statistically significant differences between subgroups (utilities for education levels not tabulated) (Table 3). In addition, preferences did not differ significantly between individuals who had knowledge about leukaemia versus those who were largely unfamiliar with the disease.

**Table 2:** Mean utility values in England and Scotland.

	England (N = 59)	Scotland (N = 30)	
Health State	Mean ± SD	Mean ± SD	Difference (*p*)
Complete Response	0.90 ± 0.12	0.92 ± 0.08	0.39

Partial Response	0.83 ± 0.16	0.87 ± 0.09	0.18

No Change	0.76 ± 0.15	0.83 ± 0.11	0.02

Progressive Disease	0.64 ± 0.21	0.75 ± 0.17	0.01

NC + 1-2 Nausea	0.70 ± 0.17	0.78 ± 0.16	0.04

NC + 1-2 Nausea/Vomiting	0.70 ± 0.16	0.78 ± 0.15	0.03

NC + 1-2 Diarrhea	0.68 ± 0.19	0.75 ± 0.17	0.07

NC + 3-4 Anemia	0.66 ± 0.19	0.74 ± 0.17	0.04

NC + 3-4 Pyrexia	0.64 ± 0.17	0.72 ± 0.17	0.03

NC + 3-4 Pneumonia	0.56 ± 0.20	0.63 ± 0.19	0.12

Second-line Treatment	0.68 ± 0.18	0.76 ± 0.13	0.02

Third-line Treatment	0.61 ± 0.24	0.72 ± 0.16	0.01

**Table 3:** Comparisons of mean utilities among subgroups.

	Age		Sex		Knowledgeable about leukaemia	
	**<60 years** **(N = 63)**	**≥60 years** **(N = 26)**	**Male** **(N = 39)**	**Female** **(N = 50)**	**Yes** **(N = 23)**	**No** **(N = 66)**
**Health State**	**Mean ± SD**	**Mean ± SD**	**Mean ± SD**	**Mean ± SD**	**Mean ± SD**	**Mean ± SD**

Complete Response	0.92 ± 0.09	0.88 ± 0.13	0.92 ± 0.08	0.89 ± 0.12	0.90 ± 0.13	0.91 ± 0.10

Partial Response	0.84 ± 0.14	0.84 ± 0.14	0.87 ± 0.10	0.82 ± 0.16	0.83 ± 0.17	0.85 ± 0.13

No Change	0.78 ± 0.14	0.80 ± 0.16	0.80 ± 0.13	0.77 ± 0.15	0.78 ± 0.17	0.79 ± 0.14

Progressive Disease	0.69 ± 0.19	0.65 ± 0.23	0.69 ± 0.18	0.67 ± 0.22	0.67 ± 0.23	0.68 ± 0.19

NC + 1-2 Nausea	0.72 ± 0.19	0.74 ± 0.12	0.73 ± 0.14	0.72 ± 0.19	0.76 ± 0.15	0.72 ± 0.18

NC + 1-2 Nausea/Vomiting	0.73 ± 0.16	0.73 ± 0.15	0.75 ± 0.14	0.71 ± 0.17	0.71 ± 0.15	0.73 ± 0.16

NC + 1-2 Diarrhea	0.70 ± 0.21	0.71 ± 0.14	0.70 ± 0.18	0.71 ± 0.20	0.67 ± 0.19	0.71 ± 0.19

NC + 3-4 Anemia	0.70 ± 0.19	0.65 ± 0.17	0.69 ± 0.16	0.68 ± 0.20	0.72 ± 0.21	0.67 ± 0.18

NC + 3-4 Pyrexia	0.66 ± 0.18	0.68 ± 0.15	0.66 ± 0.16	0.67 ± 0.18	0.64 ± 0.14	0.67 ± 0.18

NC + 3-4 Pneumonia	0.59 ± 0.20	0.58 ± 0.17	0.59 ± 0.19	0.58 ± 0.20	0.55 ± 0.19	0.59 ± 0.20

Second-line Treatment	0.71 ± 0.17	0.72 ± 0.16	0.74 ± 0.15	0.69 ± 0.18	0.69 ± 0.18	0.72 ± 0.16

Third-line Treatment	0.65 ± 0.23	0.63 ± 0.20	0.66 ± 0.17	0.64 ± 0.26	0.63 ± 0.24	0.65 ± 0.21

Note: No differences within subgroups statistically significant (p < 0.05; Student's t test)

## Discussion

This study yielded general population utilities for a universal set of CLL health states. In future studies, these utilities can be useful in the evaluation of CLL treatments from the general public perspective and can be applied to clinical trial data. Specifically, one can map the health status of individual patients from a clinical trial to the health states from this study, assign the corresponding utility weights, and use these to compute quality-adjusted life expectancy [23]. The use of the vignette approach, as opposed to a generic utility assessment, made it possible to gauge the impact of knowledge of clinical response as well as potential CLL treatment toxicities on individual preferences. As was expected, preferences for the health states decreased with reduced treatment responsiveness and with increasing grade of treatment-related toxicity.

There are similarities between the utilities obtained in this study and those estimated by the Wessex Committee, which were intended for use in a study of fludarabine relative to cyclophosphamide plus doxorubicin plus prednisolone (CAP) [11]. Specifically, the Wessex Committee estimated that the "QoL with disease" state would have a utility of 0.81, and in this study the utility for the "no change" health state was 0.78. The utility in this study for "complete response" was slightly lower than that estimated by Wessex for "QoL in remission" (0.91 vs. 0.96), which may be expected given that the vignettes used in this study included the knowledge that one has 'cancer of the blood' regardless of the existence of symptoms.

Previously, Lloyd et al [24] and Beusterien et al [25] used standard gamble to obtain general population utilities for health states experienced in breast cancer and advanced melanoma, respectively. Both studies found a difference of +0.08 between the stable disease and partial response health states. Similarly, the difference between stable disease and partial response in this study was +0.07. Given that, across the three studies, the only difference between these health states is that one is responding to treatment, this suggests that the value of hope is equivalent to +0.07 or +0.08 points on a 0-1.0 utility scale. Szabo and colleagues used the time trade-off technique to elicit utilities from the general population in different countries for health states reflecting 'responding to treatment' and 'not responding to treatment' for the chronic, accelerated, and blast phases of chronic myelogenous leukaemia [26]. Within each of these phases, the differences in mean utility values between responders versus non-responders ranged from 0.18 to 0.25. This approximates the difference of 0.23 between complete response and progressive disease observed in this study.

We attempted to recruit respondents according to the distributions of age and sex of the target populations in England and Scotland. We had a slightly lower percentage of respondents from 18-24 years of age (8% vs. 13%) and a slightly higher percentage of respondents who were in the older age group (> 60 years of age) (29% vs. 26%). In addition, the study sample had attained higher levels of education relative to the general population. Given that our study did not find age or sex to be predictors of utility-based preferences, it is unlikely that we would have observed different results with a more representative sample. Moreover, differences in respondent age or other demographic variables have not previously been shown to be reliable predictors of health state utilities [27].

Respondents in Scotland had slightly higher utilities across the health states relative to those in England. The reason for this difference in preferences is unknown, but may be attributable to cultural factors. Observing regional differences in utility weights is consistent with findings from previous research. For example, studies using the EQ-5D have shown substantial inter-country differences in utilities, including studies focusing on patients with cancer [14,28-30]. In addition, a few direct estimation studies have identified systematic differences between countries in utility weight estimates [31]. Nevertheless, despite the differences observed between England and Scotland, the relative differences in utilities among the health states generally were comparable, resulting in the utility decrements associated with toxicities to be about the same between regions.

We intentionally did not include a health state domain focusing on how worried the patient would be in the health state. Instead, we allowed the respondents to weigh their own emotional reactions to the health states. Preferences for health states can vary substantially across individuals, and subjects may vary considerably in their emotional reactions. This variability in emotional impacts was demonstrated by Bertero *et al *[13], who found that some patients with chronic leukaemia may remain positive, and others may not cope well with the knowledge of their cancer. Moreover, being on treatment may have a positive emotional benefit. For example, a large web-based survey found that CLL patients who were previously treated or who were on active treatment reported the same or higher scores on social/family, emotional well-being, and overall QoL scales relative to untreated patients [3]. For this CLL study, we did not wish to impose levels of worry on each of the health states; instead, the emotional burden associated with the health state would be embodied in the resulting utility values.

In addition, our study did not consider health states with multiple toxicities. Several recent studies have explored the estimation of utilities given this scenario. Dale *et al *[32] and Fu and Kattan [33] recommend using a minimum model, which predicts a joint-state utility as equal to the lower of the two given single-state utilities for an individual.

We believe we have conducted a rigorous study eliciting utilities for CLL health states using the widely used standard gamble approach and assessing the perspective of individuals from the general population. Utility techniques mainly vary in that the values can be obtained by individuals currently experiencing the state of interest versus indirectly via a description of that state, called a vignette, as was performed in this study. With respect to the use of vignettes, the valuation might come from patients, clinicians or the public; and the health state description might come from qualitative research with patients, condition-specific patient reported outcome measures or a descriptive state classification system like the EQ-5D or Health Utilities Index (HUI) [34]. Because general health status measures like the EQ-5D or HUI are generic, they may not adequately capture key psychological impacts associated with CLL treatment, for example, the impact of knowing that one is responding to treatment. For example, decreases in quality of life during interferon-α treatment for advanced melanoma were offset by the reduced risk of recurrence and mortality when vignette-based utilities were applied [35,36]. In contrast, IFN was only marginally better in treating melanoma than best supportive care, when the generic EQ-5D utilities were employed based on prospectively collected data [37].

Although HTA agencies prefer that utility data from generic measures be used in cost-effectiveness analyses, they acknowledge that such data may not be applicable or available. In such cases, the SMC is willing to accept data from other sources including surveys involving "direct measurement of utilities for appropriate disease/condition health states. This should use time trade-off or standard gamble methods of utility elicitation"[5]. NICE acknowledges that utilities can be elicited using the standard gamble or time trade-off approach to value specially constructed vignettes in situations where there is no reference case data or it is felt that the standardized measures do not capture all relevant aspects of the condition or its treatment. However, they state that the problem with this approach is that the vignettes are not directly linked to trial evidence, raising doubts about their validity in cost effectiveness evaluations [4].

Although the utility data in this study are not based on a measure used in conjunction with a trial in CLL, we believe that one should be able to map the health states or vignettes in this study directly to those observed in such trials. Specifically, the descriptions of treatment response within the health states were based on the standard clinical criteria used to classify treatment efficacy in CLL clinical trials [20]. With respect to the toxicities that can occur, we believe that application of utilities obtained for the toxicity vignettes in this study may even be more accurate than those based on a generic measure used in a trial, particularly because it may be difficult to administer a patient reported outcomes measure in a timely way in conjunction with such events. Given the standardized descriptions used for the health states in this study, we believe that they could mirror Markov states to which clinical trial patients could be assigned.

## Conclusions

The study reports general population health state utilities from the UK, including both England and Scotland, for a universal set of CLL states, including potential clinical outcomes and toxicities associated with various treatments. This study employed a rigorous process for the development of standardized health states that incorporated both intended treatment responses and unintended events. The utilities generated in this study can be applied in future cost-utility analyses of treatments for CLL.

## Competing interests

This study was sponsored by Napp Pharmaceuticals, Limited.

## Authors' contributions

KMB, JG, AO, and SBJ participated in the conceptualization, design, and execution of the study. KMB and JG performed the statistical analysis and drafted the manuscript.

JD, ML, and DM provided clinical expertise to inform the content of the health states valuated in the study. All authors read and approved the final manuscript.

## Supplementary Material

Additional file 1 **CLL Health States**. This file contains the complete set of health state descriptions that participants valuated in the study. These include four CLL treatment-related response states, six toxicity-related health states, and two health states reflecting the impact of undergoing a second or third course of treatment. Click here for file
