# Supplementary material for: Population preference values for treatment outcomes in chronic lymphocytic leukaemia: a cross-sectional utility study
Source: Health Qual Life Outcomes. 2010 May 18;8:50. doi: 10.1186/1477-7525-8-50 (PMC2890699; doi:10.1186/1477-7525-8-50)
Supplement: Additional file 1 — CLL Health States. This file contains the complete set of health state descriptions that participants valuated in the study. These include four CLL treatment-related response states, six toxicity-related health states, and two health states reflecting the impact of undergoing a second or third course of treatment. [file 1477-7525-8-50-S1.DOCX]

CLL Health States (*symbols, rather than titles, were used to indicate the health states during the interviews)*

*Complete Response (CR)*

• You have cancer of the blood that may not be life-threatening, but it may affect your well-being. This disease results in a weakened immune system, increasing the chance of getting infections.

• Your symptoms have improved with treatment.

• You do not have swollen glands in your neck, armpits, or groin.

• Daily activities do not take more effort than usual, but you feel slightly fatigued (tired and weak). You do not feel short of breath during normal activities.

• Your appetite is normal.

• You do not have trouble sleeping because of night sweats.

*Partial Response (PR)*

• You have cancer of the blood that may not be life-threatening, but it may affect your well-being. This disease results in a weakened immune system, increasing the chance of getting infections.

• Your symptoms have improved with treatment.

• Your swollen glands in your neck, armpits, or groin are smaller than they were before treatment.

• Daily activities may take more effort than usual, and you feel a little fatigued (tired and weak). You may feel short of breath during normal activities.

• Sometimes, you don’t feel hungry or you feel full after eating a little.

• You occasionally have trouble sleeping because of night sweats that wake you up.

*No Change (NC)*

• You have cancer of the blood that may not be life-threatening, but it may affect your well-being.  This disease results in a weakened immune system, increasing the chance of getting infections.

• Your symptoms have stabilized with treatment. Your symptoms have not improved or worsened.

• You have swollen glands in your neck, armpits, or groin that look like lumps and may be visible and uncomfortable.

• Daily activities take more effort than usual, and you often are fatigued (tired and weak). You feel short of breath during normal activities.

• Much of the time, you don’t feel hungry or you feel full after eating a little.

• You often have trouble sleeping because of night sweats that wake you up.

*Progressive Disease (PD)*

• You have cancer of the blood that may not be life-threatening, but it may affect your well-being. This disease results in a weakened immune system, increasing the chance of getting infections.

• Your symptoms are getting worse.

• Your swollen glands in your neck, armpits, or groin are bigger and visible. They may be uncomfortable.

• Daily activities require a lot of effort, and you are almost always fatigued (tired and weak). You feel short of breath during normal activities.

• Almost always, you don’t feel hungry or you feel full after eating a little.

• Most of the time, you have trouble sleeping because of night sweats that wake you up.

*NC + nausea (grade 1 or 2)*

• You have cancer of the blood that may not be life-threatening, but it may affect your well-being. This disease results in a weakened immune system, increasing the chance of getting infections.

• Your symptoms have stabilized with treatment. Your symptoms have not improved or worsened.

• You have swollen glands in your neck, armpits, or groin that look like lumps and may be visible and uncomfortable.

• Daily activities take more effort than usual, and you often are fatigued (tired and weak). You feel short of breath during normal activities.

• Much of the time, you don’t feel hungry or you feel full after eating a little.

• You often have trouble sleeping because of night sweats that wake you up.

• Once a month when you receive treatment, you experience nausea for 24-48 hours, during which time you don’t feel like eating, and food may have a funny metallic taste. Most of the time, this can be relieved with medication.

*NC + nausea/vomiting (grade 1 or 2)*

• You have cancer of the blood that may not be life-threatening, but it may affect your well-being. This disease results in a weakened immune system, increasing the chance of getting infections.

• Your symptoms have stabilized with treatment. Your symptoms have not improved or worsened.

• You have swollen glands in your neck, armpits, or groin that look like lumps and may be visible and uncomfortable.

• Daily activities take more effort than usual, and you often are fatigued (tired and weak). You feel short of breath during normal activities.

• Much of the time, you don’t feel hungry or you feel full after eating a little.

• You often have trouble sleeping because of night sweats that wake you up.

• Once a month when you receive treatment, you experience nausea and vomiting for 24-48 hours, during which time you don’t feel like eating, and food may have a funny metallic taste. Most of the time, this can be relieved with medication.

*NC + diarrhea (grade 1 or 2)*

• You have cancer of the blood that may not be life-threatening, but it may affect your well-being.  This disease results in a weakened immune system, increasing the chance of getting infections.

• Your symptoms have stabilized with treatment. Your symptoms have not improved or worsened.

• You have swollen glands in your neck, armpits, or groin that look like lumps and may be visible and uncomfortable.

• Daily activities take more effort than usual, and you often are fatigued (tired and weak). You feel short of breath during normal activities.

• Much of the time, you don’t feel hungry or you feel full after eating a little.

• You often have trouble sleeping because of night sweats that wake you up.

• Once a month when you receive treatment, you experience 3 to 4 episodes per day of diarrhea (watery stools) that lasts for 3 to 4 days.

*NC + anemia (grade 3 or 4)*

• You have cancer of the blood that may not be life-threatening, but it may affect your well-being.  This disease results in a weakened immune system, increasing the chance of getting infections.

• Your symptoms have stabilized with treatment. Your symptoms have not improved or worsened.

• You have swollen glands in your neck, armpits, or groin that look like lumps and may be visible and uncomfortable.

• You experience substantial fatigue (tiredness/weakness), and your ability to exercise (walking or shopping, etc.) is substantially limited. You feel short of breath during normal activities. You receive a 6-hour blood transfusion at the clinic, which relieves the fatigue for 2-3 weeks.

• Much of the time, you don’t feel hungry or you feel full after eating a little.

• You often have trouble sleeping because of night sweats that wake you up.

*NC + pyrexia (grade 3 or 4)*

• You have cancer of the blood that may not be life-threatening, but it may affect your well-being.  This disease results in a weakened immune system, increasing the chance of getting infections.

• Your symptoms have stabilized with treatment. Your symptoms have not improved or worsened.

• You have swollen glands in your neck, armpits, or groin that look like lumps and may be visible and uncomfortable.

• Daily activities take more effort than usual, and you often are fatigued (tired and weak). You feel short of breath during normal activities.

• Much of the time, you don’t feel hungry or you feel full after eating a little.

• You often have trouble sleeping because of night sweats that wake you up.

• Once a month, you develop a fever due to infection, and this requires treatment in the hospital for 4 to 5 days.

*NC + pneumonia (grade 3 or 4)*

• You have cancer of the blood that may not be life-threatening, but it may affect your well-being.  This disease results in a weakened immune system, increasing the chance of getting infections.

• Your symptoms have stabilized with treatment. Your symptoms have not improved or worsened.

• You have swollen glands in your neck, armpits, or groin that look like lumps and may be visible and uncomfortable.

• Daily activities take more effort than usual, and you often are fatigued (tired and weak). You feel short of breath during normal activities.

• Much of the time, you don’t feel hungry or you feel full after eating a little.

• You often have trouble sleeping because of night sweats that wake you up.

• Once a month, you have pneumonia, which causes coughing, chest pain, fever, and breathlessness. This requires you to stay in the hospital for 7-10 days and receive intravenous antibiotics.

*Second-line treatment*

• You have cancer of the blood that may not be life-threatening, but it may affect your well-being.  This disease results in a weakened immune system, increasing the chance of getting infections.

• This is your second time on treatment because your symptoms worsened.

• You have swollen glands in your neck, armpits, or groin that look like lumps and may be visible and uncomfortable.

• Daily activities require quite a bit of effort, and you are fatigued (tired and weak) much of the time. You feel short of breath during normal activities.

• Much of the time, you don’t feel hungry or you feel full after eating a little.

• You often have trouble sleeping because of night sweats that wake you up.

*Third-line treatment*

• You have cancer of the blood that may not be life-threatening, but it may affect your well-being.  This disease results in a weakened immune system, increasing the chance of getting infections.

• This is your third time on treatment because your symptoms worsened.

• You have swollen glands in your neck, armpits, or groin that look like lumps and may be visible and uncomfortable.

• Daily activities require a lot of effort, and you are almost always fatigued (tired and weak). You feel short of breath during normal activities.

• Much of the time, you don’t feel hungry or you feel full after eating a little.

• You often have trouble sleeping because of night sweats that wake you up.
